# Development and testing of an endoscopic pseudo-viewpoint alternating system

**DOI:** 10.1007/s11548-014-1083-z

**Published:** 2014-06-21

**Authors:** Y. Koreeda, S. Obata, Y. Nishio, S. Miura, Y. Kobayashi, K. Kawamura, R. Souzaki, S. Ieiri, M. Hashizume, M. G. Fujie

**Affiliations:** 1 Graduate School of Science and Engineering, Waseda University, 59-309, 3-4-1, Okubo, Shinjuku Ward, Tokyo, Japan; 2 Center for the Integration of Advanced Medicine and Innovative Technology, Kyushu University, Fukuoka, Japan; 3 Faculty of Engineering, Chiba University, Chiba, Japan; 4 Faculty of Science and Engineering, Waseda University, Tokyo, Japan

**Keywords:** Minimally invasive surgery, Image processing, Endoscope, Depth perception, Virtual reality

## Abstract

**Purpose:**

An endoscopic system is needed that presents informative images irrespective of the surgical situation and the number of degrees of freedom in endoscopic manipulation. This goal may be achieved with a virtual reality view for a region of interest from an arbitrary viewpoint. An endoscopic pseudo-viewpoint alternation system for this purpose was developed and tested.

**Method:**

Surgical experts and trainees from an endoscopic surgery training course at the minimally invasive surgery training center of Kyushu University were enrolled in a trial of a virtual reality system. The initial viewpoint was positioned to approximate the horizontal view often seen in laparoscopic surgery, with $20^{\circ }$ between the optical axis of the endoscope and the task surface. A right-to-left suturing task with right hand, based on a task from the endoscopic surgery training course, was selected for testing. We compared task outcomes with and without use of a new virtual reality-viewing system.

**Result:**

There was a 0.37 mm reduction in total error ($p = 0.02$) with use of the proposed system. Error reduction was composed of 0.1 mm reduction on the y-axis and 0.27 mm reduction on the x-axis. Experts benefited more than novices from use of the proposed system. Most subjects worked at a pseudo-viewpoint of around 34$^\circ$.

**Discussion:**

Suturing performance improved with the new virtual reality endoscopic display system. Viewpoint alternation resulted in an overview that improved depth perception and allowed subjects to better aim the marker. This suggests the proposed method offers users better visualization and control in endoscopic surgery.

## Introduction

### Background

Minimally invasive surgery is attracting attention today. One of the most utilized forms of minimally invasive surgery is laparoscopy, which is carried out by placing multiple trocars through incisions on the abdominal wall and by inserting forceps and an endoscope on the inflated abdominal cavity through the trocars. Surgeons carry out a range of medical treatments under an endoscopic view displayed on a monitor. Laparoscopy is applied to wide range of surgeries including cholecystectomy, prostatectomy and gastrostomy.

Laparoscopy requires smaller incisions as compared to conventional surgeries, which lead to fewer traumas, less blood loss, better cosmetics and a shorter stay at hospital [1]. These advantages make laparoscopic surgery an attractive alternative for patients. However, laparoscopic surgery is more demanding of surgeon skill because of counter-intuitive control of forceps and limited tactile information. Problems associated with vision, which include narrow field of view, loss of depth perception and limited positioning of the viewpoint, also challenge surgeon skill [2].

All six degrees of freedom (DoFs) are needed at the tip of an endoscope to position the viewpoint freely. However, owing to the constraints of the trocar, only four DoFs are available with a normal endoscope. Of these four DoFs, two are rotational around the trocar, one is translational along the shaft of the endoscope and one is rotational around the shaft of the endoscope. The rotational DoF around the shaft of the endoscope is not often used because the direction of gravity must remain constant [3]. This means that an endoscope has only three useful DoFs. Therefore, in laparoscopic surgery, there is only one orientation an endoscope can be given when a surgeon is trying to observe a region of interest.

It is sometimes difficult to position the viewpoint well enough to provide the required information about an intervention site. In that case, the surgeon must reconstruct the view from his/her insight to understand the current state of the site. Since view reconstruction of the intervention site can be difficult, the reconstructed view may be incorrect in some important details.

### Motivation

The limited positioning of the viewpoint due to insufficient DoFs of an endoscope forces surgeons to work with their insight. This is a problem because perception through an endoscopic view depends on surgeon skill, which increases the risk of a failure in understanding. The motivation for our study was to expand the reachable viewpoints in laparoscopy.

Exchanging straight-viewing endoscope and oblique-viewing endoscope is frequently seen in laparoscopy, allowing surgeons to observe surgical region of interest from a different viewpoint. More reachable viewpoints can be achieved by increasing DoFs at the tip of an endoscope. ENDOCAMELEON$^{\textregistered }$ (Karl Storz & Co. KG, Tuttlingen, Germany), for example, adds one bending DoF to the tip of an endoscope. This allows switching between an overlooking view and a horizontal view. Another example is a snake like endoscope with 7 DoFs, which allows a wide range of orientations near the region of interest [4].

Approaches that employ hardware configurations as mentioned above allow observation of the region of interest from more than one direction without losing superiority of using a rigid endoscope; i.e., high resolution and low latency. However, the approaches that employ hardware configurations lack versatility because reachable viewpoints depend on the configuration of DoFs and on the surgical situation. To ensure an arbitrary orientation in relation to the region of interest, an endoscope requires more than standard set of four DoFs. The number required for an arbitrary viewpoint cannot be predicted because surgical conditions change according to patient body shape, patient diagnosis and surgeon preferences, for example, thus, there are innumerable combinations of conditions. Any increase in DoFs of an endoscope leads to an increase in endoscope diameter, more complicated control mechanisms and higher cost. Because an increase in DoFs by use of hardware is not an optimum solution and that it does not prevent collision of endoscope and tissues, a viewpoint orientation control that does not rely on a mechanical configuration is preferable.

**Fig. 1:**
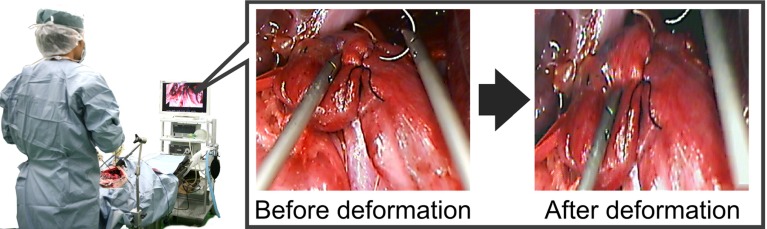
Pseudo-viewpoint alternating system. The system takes images from endoscopes and presents them to surgeon as images from another viewpoint

To allow non-mechanical vision control, we developed pseudo-viewpoint alternation by use of image processing (Fig. 1). An image from an endoscope viewpoint was processed into image from another viewpoint. Our goal was to develop an endoscopic system that bypasses physical limitations by presenting virtual images of the region of interest as generated from a different viewpoint.

A concept of presenting virtual images as generated from a different viewpoint was proposed by Breedveld et al. [5]. Wentink et al. [6] conducted evaluation of [5], which was limited in that evaluation was carried out by physically rotating monitor along its normal. The concept was not actually implemented by image processing, evaluating only the effect of rotational viewpoint alternation along the endoscope’s shaft, which was clinically unrealistic.

Koppel et al. [7] presented viewpoint alternation method using image processing. In this study, viewpoint alternation was implemented and was verified in terms of precision. However, this study did not take considerations of how a surgeon interferes with the system. An interface for surgeon to interactively control viewpoint was not implemented and effects of viewpoint alternation on surgical tasks were not evaluated.

In this paper, we report viewpoint alternation system with interactive control interface and evaluation of the pseudo-viewpoint alternation method on surgical tasks. In second section, we explain the abstract scheme of an image processing method that applies “pseudo-viewpoint alternation” to images obtained from an endoscope. In third section, we describe the method of a quantitative performance assessment and materials used to implement proposed method. The performance assessment was carried out by comparing task performances with and without the pseudo-viewpoint alternating system. The “Result” of experiment is presented in fourth section and “Discussion” in fifth section.

## Pseudo-viewpoint alternation

**Fig. 2:**
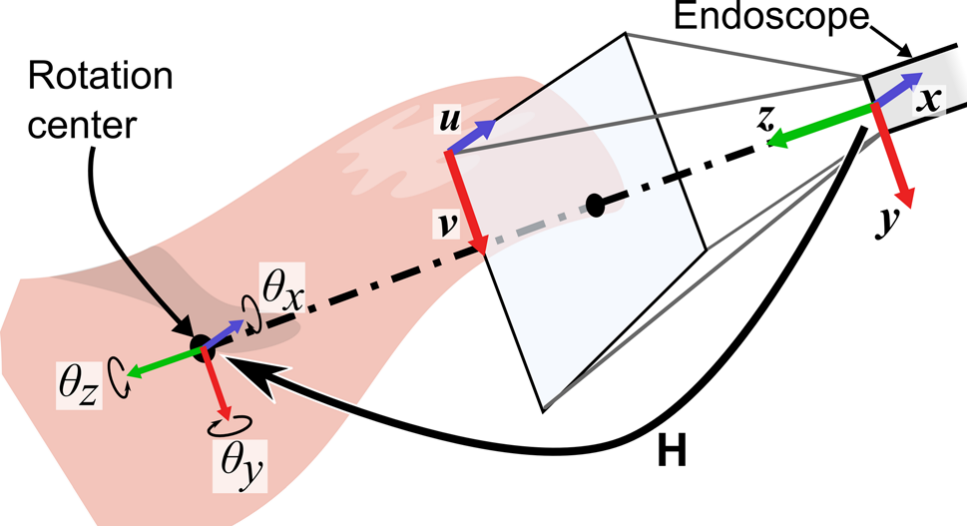
The coordinate system. Coordinate origin is at the center of endoscope. ${\varvec{{u}}}$ and ${\varvec{{v}}}$ are defined on the projection surface of the pinhole hole camera model. **H** is the rotation center and $\theta _i$ is defined as rotation around $i$ axis

**Fig. 3:**
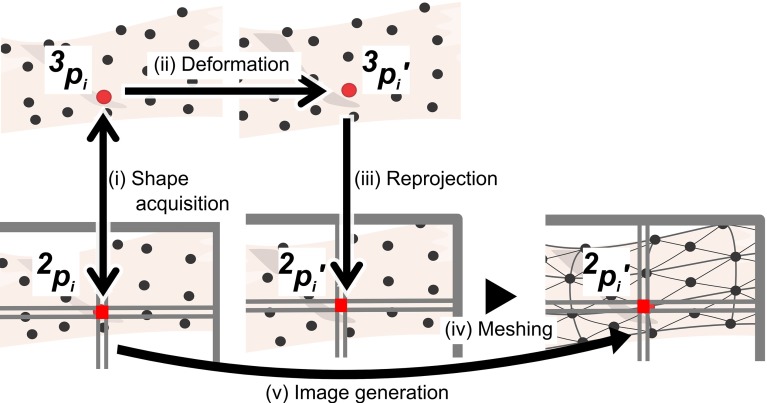
System flow. pseudo-viewpoint alternation involves a series of coordinate transformations

The pseudo-viewpoint alternating system consists of a 3D endoscope, a controller and an image processing unit. The coordinate system is illustrated in Fig. 2. The generalized system flow is as follows (Fig. 3):

### Camera calibration

A pinhole camera model is utilized in camera calibration. The endoscope is calibrated to obtain intrinsic parameters and distortion coefficients [8, 9].

Images are deformed to eliminate distortion [9]. 3D coordinates $${}^3p=\left[ {{\begin{array}{l@{\quad }l@{\quad }l} x&{} y&{} z \\ \end{array} }} \right] ^{T}$$ are projected onto image coordinates $${ }^2p=\left[ {{\begin{array}{l@{\quad }l} u&{} v \\ \end{array} }} \right] ^{T}$$ by (1) and (2) after deformation.

1 $$\begin{aligned} u&\leftarrow \frac{f_x }{z}x+u_0\end{aligned}$$

2 $$\begin{aligned} v&\leftarrow \frac{f_y }{z}y+v_0 \end{aligned}$$

$$f_x $$ and $$f_y $$ refer to the focal length of the x and y axes respectively. $$\left[ {{\begin{array}{l@{\quad }l} {u_0 }&{} {v_0 } \\ \end{array} }} \right] ^{T}$$ refers to the principal camera point.

### 3D shape acquisition

A 3D field of view is required to apply projection deformation to the images. An acquired 3D point ${ }^3p$ is associated with a pixel ${ }^2p$. 3D shape acquisition was not implemented in this paper for an experimental reason. The substitute of 3D shape acquisition is explained in “Evaluation method” section.

### Control input

The region of interest must remain in the field of view. This implies that rotation around the region of interest is the ideal control scheme for the viewpoint.

A joystick with two analog outputs was implemented as a controller. Input vector $q\in \mathbf{R}^{2}$ was mapped to the rotation angle vector $\varvec{\Delta \!\uptheta }$ by (3) and (4).

3 $$\begin{aligned}&\varvec{\Delta \!\uptheta }=\left[ \begin{array}{l@{\quad }l@{\quad }l} {\Delta \theta _x }&{} {\Delta \theta _y }&{} {\Delta \theta _z } \\ \end{array} \right] ^{T}\leftarrow \Omega \cdot \left( {q+q_0 } \right) \end{aligned}$$

4 $$\begin{aligned}&\Omega =\left[ \begin{array}{c@{\quad }c@{\quad }c} {\cos \gamma }&{} {\sin \gamma } \\ {\sin \gamma }&{} {\cos \gamma } \\ 0&{} 0 \\ \end{array} \right] \end{aligned}$$

$$q_0 $$ is a neutral position vector that is automatically set every time the program is run. The mean average of $$q$$ from 10 frames of data is set as $$q_0 $$. $$\Omega $$ is a matrix that maps the input value to the rotational vector $$\varvec{\Delta \!\uptheta }$$. $$\gamma $$ determines correspondence between joystick input direction and the direction of rotation of viewpoint.

The input rotation vector $\varvec{\Delta \!\uptheta }$ incrementally updates the position of the viewpoint. The alternation angle of the viewpoint from the origin is calculated every processing loop by (5).

5 $$\begin{aligned} \theta _i \leftarrow \theta _i +\alpha _i \Delta \theta _i^r \end{aligned}$$

$\alpha _i$ is a linear coefficient that defines how much the viewpoint reacts to controller input. $r$ is a power coefficient that allows switching between fine movement near the target and fast-forwarding movement. At $r$ = 1, the viewpoint movement is linear and no switching occurs. As $r$ increases, alternations of the viewpoint become smaller near the variables’ origin compared to near the variables’ limits.

### Points deformation according to angle input

Assuming the origin is the center of endoscope, 3D point ${}^3p^{\prime }$ in a new viewpoint coordinate can be expressed as (6).

6 $$\begin{aligned} { }^3P^{\prime }\leftarrow \mathrm{H}^{-1}\cdot R_z (\theta _z )R_y (\theta _y )R_x (\theta _x )\mathrm{H}\cdot ^3P \end{aligned}$$

${ }^3P$ is a homogenous vector of ${ }^3p$. $\mathrm{H}$ is the orientation of the endoscope in relative to the rotation center. $R_i (\theta _i )$ gives an Euler rotation matrix about the $i$ axis. Because the pixel coordinate ${ }^2p$ is associated with the 3D coordinate ${ }^3p,\,{ }^2p$ is now associated with the deformed 3D point ${ }^3p^{\prime }$.

### Points reprojection

Deformed points ${ }^3p^{\prime }$ are reprojected onto an image plane by (1) and (2) to get pixel position ${ }^2p^{\prime }$. The original pixel ${ }^2p$ is now associated with the corresponding pixel ${}^2p^{\prime }$.

### Meshing and image generation

The color vector of the original pixel ${ }^2p$ is copied to new pixel ${ }^2p^{\prime }$. Even the state-of-art 3D shape acquisition methods often fail to give depth information for a subset of pixels [10]. The system was designed to run with only a fraction of pixels with a valid depth value. The reprojected 2D plane was meshed into triangles by pixels with valid 3D coordinates. The meshing algorithm was based on Delaunay triangulation [11]. Delaunay triangulation was used because it gives near equilateral triangles which are suitable for an interpolation application. Also, Delaunay triangulation is computationally efficient. Pixels ${ }^2p^{\prime }$ without associated ${ }^2p$ had positions of associated original pixels interpolated using barycentric interpolation.

## Evaluation method

We conducted an *in vitro* experiment to examine the effectiveness of our system. Participants in an endoscopic surgery training course at the minimally invasive surgery training center of Kyushu University were asked to perform training tasks with and without the system. To evaluate the effectiveness of the pseudo-viewpoint alternation method, task outcomes with and without the system were compared.

A suture task in a horizontal endoscope setup was taken as a model case to evaluate the system. Loss of depth perception is problematic where the endoscope is positioned almost parallel to the working area. This type of endoscopic view is seen frequently in surgery deep inside the abdominal cavity such as in Nissen fundoplication. Uniformly spaced suturing is difficult with limited depth perception. Unevenly spaced sutures may result in postoperative complication. Suturing was chosen as the assessment task because suturing outcome reflects competence in surgical operation in general, thus frequently utilized as an assessment task [12, 13].

### Equipment

**Fig. 4:**
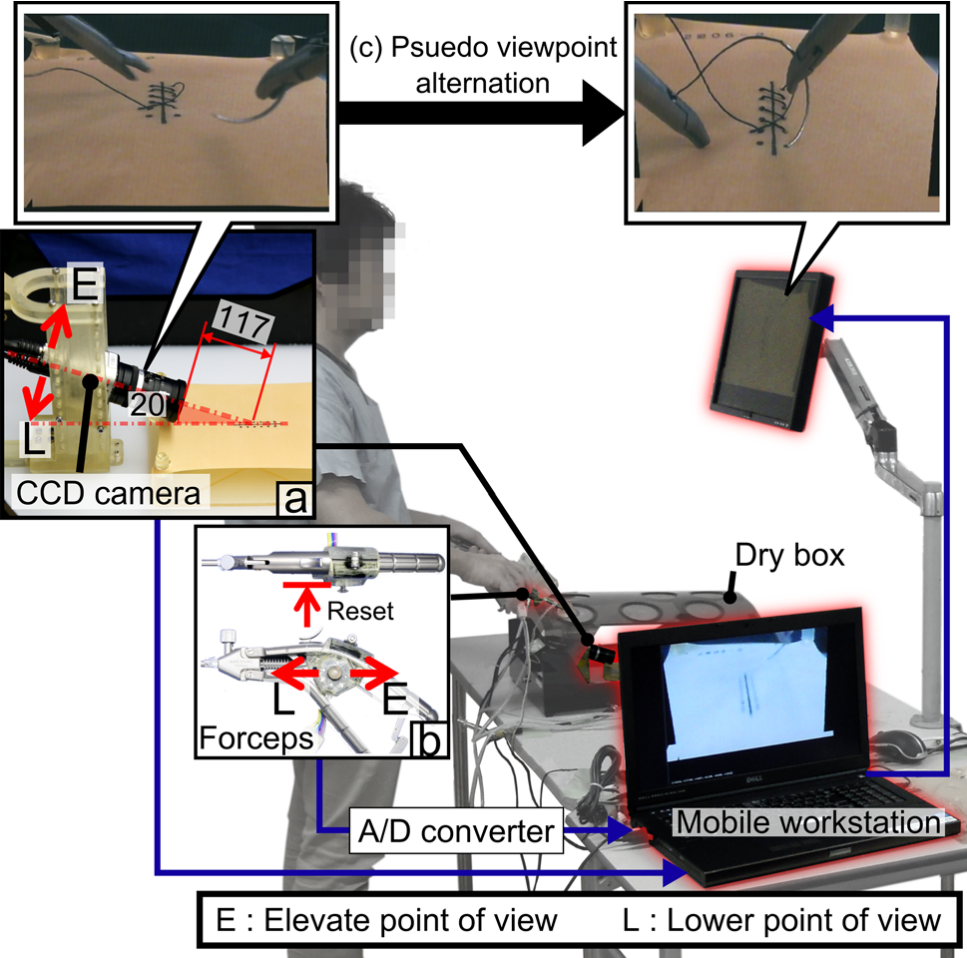
Implementation of pseudo-viewpoint alternation system for the experiment. In $(a)$, position of camera in relative to rubber sheet is shown. The camera is elevated 20 deg to rubber sheet and positioned 117 mm away. In $(b)$, joystick is positioned on the forceps’ gripping. Joystick control toward direction E/L correspond to alternation of viewpoint in elevating/lowering direction as indicated in $(a)$. $(c)$ Indicates the view alternation that is presented to the subjects

To introduce the experiment spontaneously, tasks were performed in a setup similar to an endoscopic surgery training course at the minimally invasive surgery training center (Fig. 4). A phantom was placed in the middle of dry box (M.C. Medial Inc., Tokyo, Japan). Trocars were placed on the dry box for insertion of a gun-type needle holder (Karl Storz & Co. KG, Tuttlingen, Germany).

**Fig. 5:**
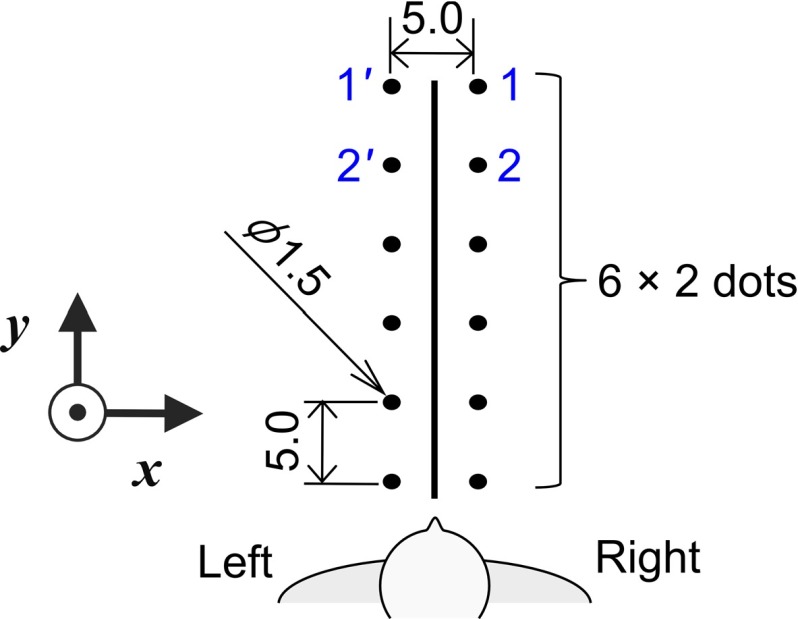
Dots printed on a rubber sheet. A number by a dot shows the suturing order. Dashed number shows a position of needle extraction

The phantom was a rubber sheet (M.C. Medical Inc., Tokyo, Japan) as shown in Fig. 5. The line represents a tissue cut and the dots show where the subjects should aim to get uniform suturing. The rubber sheet is the same as the phantom used for box training in endoscopic surgery training courses at the minimally invasive surgery training center. The y-axis was defined as the direction of the anteroposterior with subjects facing direction being positive. The x-axis was defined as the opposite direction of suturing, which corresponded to left to right in the subjects’ orientation.

A charge coupled device (CCD) camera (Watec Co., Ltd., Yamagata, Japan) with fixed focal length lens was used instead of a stereo-endoscope. This CCD camera provides similar view to an endoscope and is used in endoscopic surgery training course where the experiment was carried out. We used the CCD camera to introduce subjects to the experimental environment spontaneously. The CCD camera was positioned according to the judgment of an experienced surgeon such that it resembled the horizontal view often seen in clinical situations. The optical axis made an angle of $-20^\circ$ around x-axis with the rubber sheet.

### Implementation of pseudo-viewpoint alternation

As stated in “Pseudo-viewpoint alternation” section, 3D shape acquisition was not implemented in this paper. This was because the phantoms had no texture. Stereopsis-based depth acquisition methods utilize differentiation in color for matching, making it inapplicable on the textureless surface. Instead, a set of uniformly distributed 3D points of a phantom was provided to the system in each processing loop. Resolution of the 3D points was 0.2 sample points per mm which was equivalent to dot pitch of 5 mm.

The joystick controller was placed on the forceps grip to allow control of the pseudo-viewpoint alternation. Coefficient $\gamma$ in (4) was set such that pulling the joystick toward the palm resulted in elevation of the viewpoint to an overlooking position and pushing the joystick away from palm did the opposite. Coefficient matrices H and $\alpha _i$ in (6) and (5) were set as (7) and (8), respectively.

7 $$\begin{aligned} \mathrm{H}&= \left[ \begin{array}{l@{\quad }l@{\quad }l@{\quad }c} 1&{} 0&{} 0&{} 0 \\ 0&{} 1&{} 0&{} 0 \\ 0&{} 0&{} 1&{} {120} \\ 0&{} 0&{} 0&{} 1 \\ \end{array} \right] \end{aligned}$$

8 $$\begin{aligned} \nonumber \\ \alpha _y&= \alpha _z =0 \end{aligned}$$

Equation (7) means that rotation center of alternation of the viewpoint was at the center of phantom. (8) means that the joystick had only one DoF which controlled the camera rotation velocity about the x-axis. Parameter $r$ in (5) was set at 2. $\alpha _x$ was set such that the maximum input resulted in a rotation of 28.2$^\circ$/s. Coefficients were set according to the judgment of an experienced surgeon and were set where the surgeon felt it would be comfortable for control. In this experiment, the joystick can also be used as a push switch, which was associated with resetting $\varvec{\uptheta }$ to a zero vector.

We previously reported that the system recorded at an average frame rate of 7.0 frames per second with a maximum 1.00 mm rendering error excluding the 3D shape acquisition [14]. Frame rate and rendering error were sufficient to give a sense of reality to the alternation of viewpoint. Our new implementation recorded 16.0 frames per second in the same condition as [14].

### Procedure

Subjects were allocated randomly into groups A and B. Both groups practiced with pseudo-viewpoint alternation enabled. They were allowed to practice until they were satisfied that they understood the experimental tasks and the joystick control. No subject practiced for more than 10 min.

- 1st task: Subjects in group A started a task with pseudo-viewpoint alternation enabled and group B with it disabled.
- 2nd task: Subjects in group A then carried out a task with pseudo-viewpoint alternation disabled and group B with it enabled.

### Tasks

A suturing task in the endoscopic surgery training courses at the minimally invasive surgery training center was used. Subjects were to pick up and hold needles in their right hand. Then subjects were to suture from right to left. Subjects were to aim at the dots printed on the rubber sheet and were asked to perform sutures as precisely as possible. Cut up time was 5 min but time was extended when (a) no suture was completed or (b) the subject was engaged in suturing, i.e., the needle was in contact with the rubber sheet.

### Subjects

Nine subjects participated in the experiment. Surgical experience and the grouping of subjects are shown in Table 1. Subjects with less than 30 experiences in surgical interventions were classified as novices and otherwise experts.

**Table 1:** Surgical experiences and the grouping of the subjects

No.	Group	Classification	Specialty	No. of interventions*	
1	A	Expert	Gastroenterologist	50	(50)
2	A	Expert	Gastroenterologist	30	(40)
3	A	Expert	Pediatric	30	(70)
4	A	Novice	Pediatric	5	(15)
5	B	Expert	Pediatric	300	(300)
6	B	Expert	Pediatric	30	(70)
7	B	Expert	Pediatric	30	(50)
8	B	Novice	Pediatric	5	(10)
9	B	Novice	Pediatric	1	(9)

* Laparoscopy only. Figures in parentheses show experiences as an assistant

The aim of the experiment and the task were explained thoroughly to the subjects before they started the experiment. Subjects were assured that the data would be used in a form that revealed no personal information that would identify individuals. No physical or mental risk was present in the experiment.

## Result

**Fig. 6:**
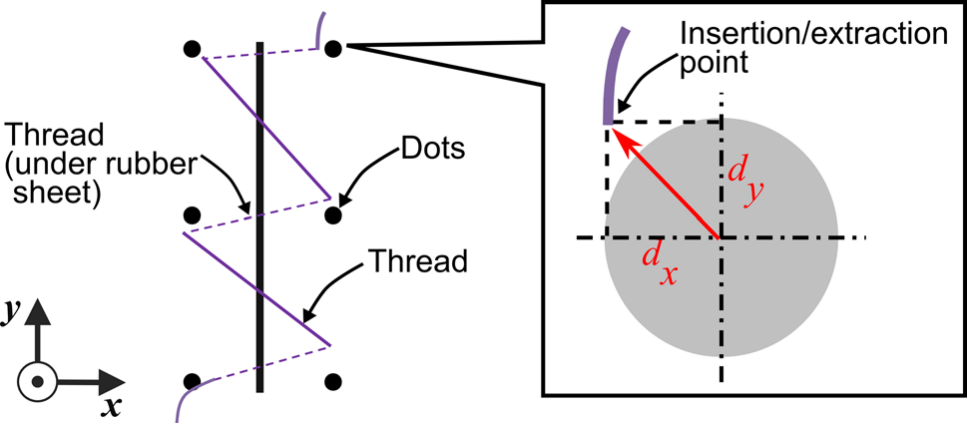
Definition of the error. Error is measured from the *center of the dots*. *Black dots* *and line* indicates the prints. *Purple line* indicate the thread

Rubber sheets were photographed with a high-resolution digital camera with a chessboard marker placed on the rubber sheet after the experiment. The images were digitally deformed by a projection transformation such that the chessboard looked uniform. This transformation was used to compensate for the horizontal slant of the camera. Points of needle insertion and extraction were identified and located manually under magnified view. A suture error was measured from the center of a round mark to the position of insertion/extraction (Fig. 6). In this paper, one suture is considered as consisting of two needlings, i.e., an insertion and an extraction.

Subjects completed at least one suture up to the maximum of six sutures. The Mann–Whitney U test was used to test the difference between conditions with level of significance set $p < 0.05$.

**Fig. 7:**
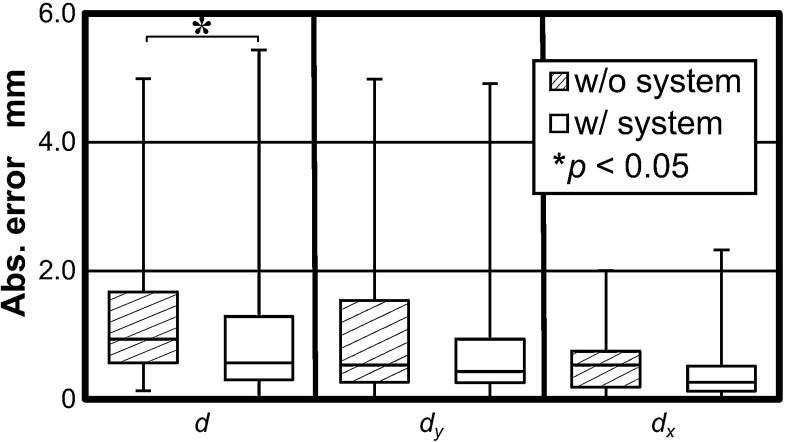
Errors with and without the system. $d$ refers to total error. $d_{x}$ and $d_{y}$ refer to error in the x and y directions, respectively

**Fig. 8:**
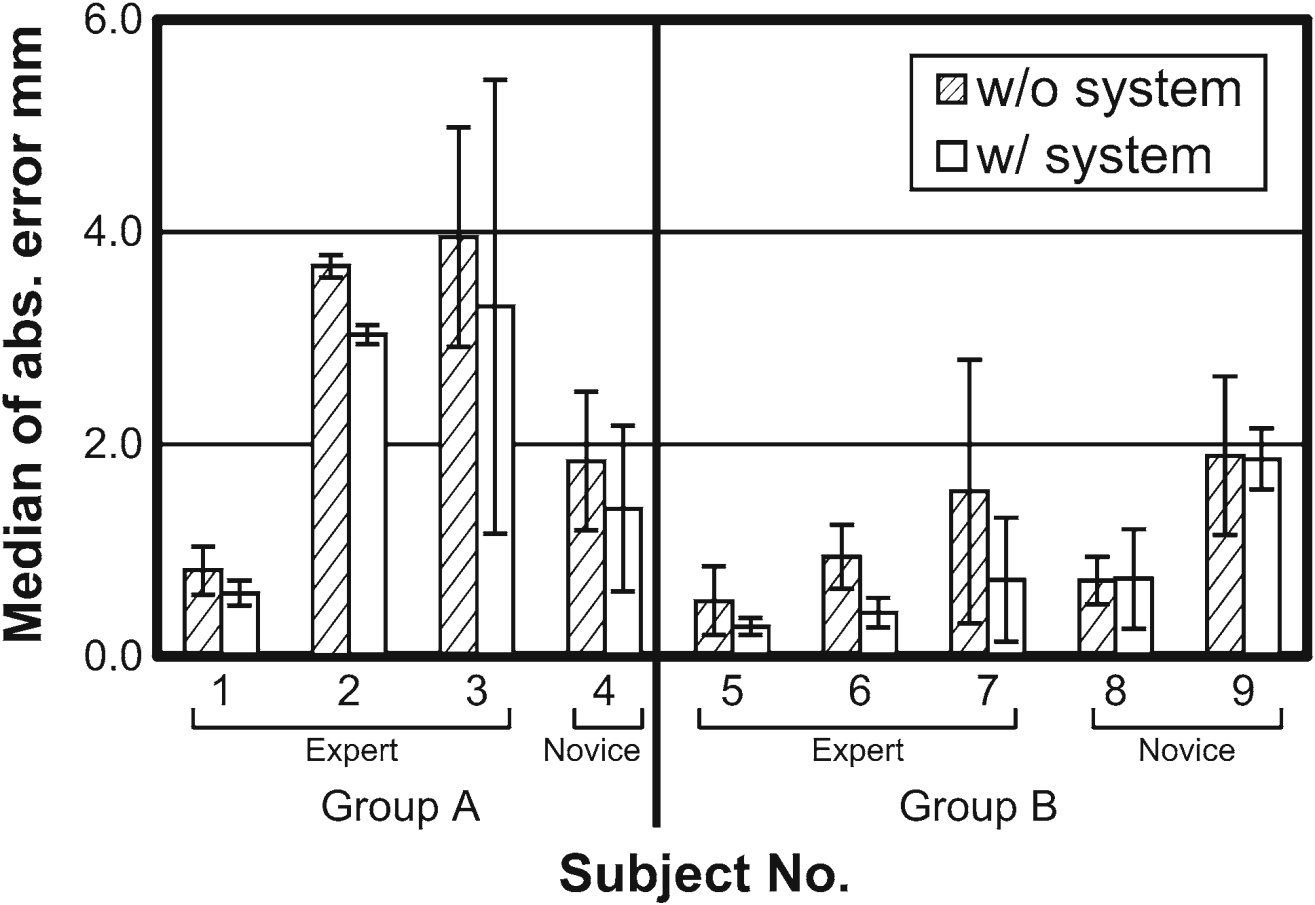
Median errors for each subject. *Error bars* show median absolute deviations

**Fig. 9:**
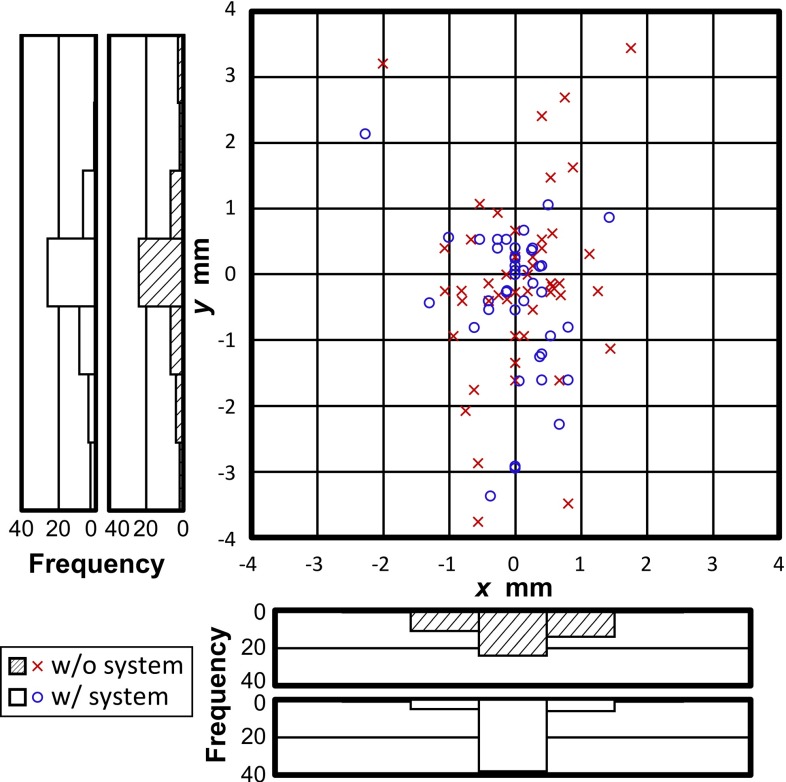
Scatter graph and histogram of needle insertion/extraction points

**Fig. 10:**
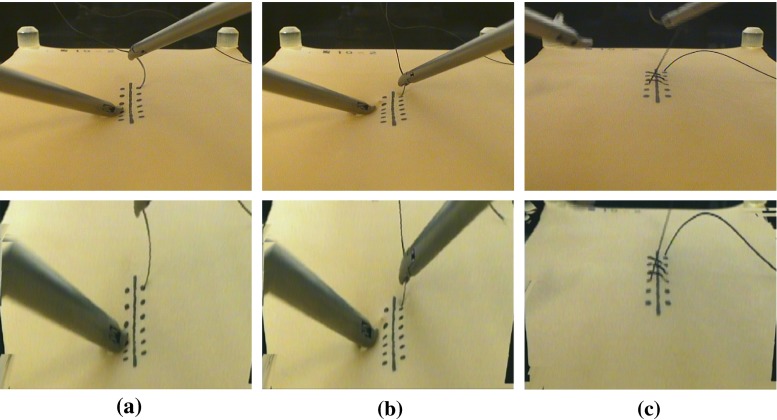
Source images and generated image. Images on the bottom row show the generated images from images on the top row, **a** insertion ($\theta _{x} = 25$), **b** extraction ($\theta _{x} =25$), **c** completed ($\theta _{x} = 15$)

Median errors for all sutures with and without pseudo-viewpoint alternation enabled are compared in Fig. 7. There is a 0.37 mm reduction in error with it enabled ($p = 0.02$). The error reduction was 0.1 mm for error on the y-axis and 0.27 mm for error on the x-axis. Subject-wise errors are shown in Fig. 8. Distributions of suture insertions/extractions are shown on Fig. 9. Distributions of suturing errors were tested with Shapiro–Wilk test. The null hypothesis, a sample is normally distributed, was rejected for both x- and y-axis in condition with the system ($p < 0.001$) for y-axis in condition without the system ($p = 0.029$) but was not rejected on x-axis ($p = 0.9$). We concluded that suturing errors were not normally distributed. Figure 10 shows few samples of generated images.

There was a notable difference in trend between experts and novices. There is a 0.65 mm reduction in median error for experts compared to a 0.16 mm reduction for novices. Error reduction in the expert class was statistically significant ($p = 0.002$) but not significant in the novice class ($p = 0.4$).

Group A, which started tasks with system enabled, had median suturing error of 1.7 mm without system and 0.92 mm with system (45 % reduction). Group B, which started tasks with system disabled, had median suturing error of 0.85 mm without system and 0.47 mm with system (45 % reduction). Inter-group comparison was statically significant in both conditions ($p = 0.009$ and $p = 0.04$ in task without system and in task with system, respectively).

**Fig. 11:**
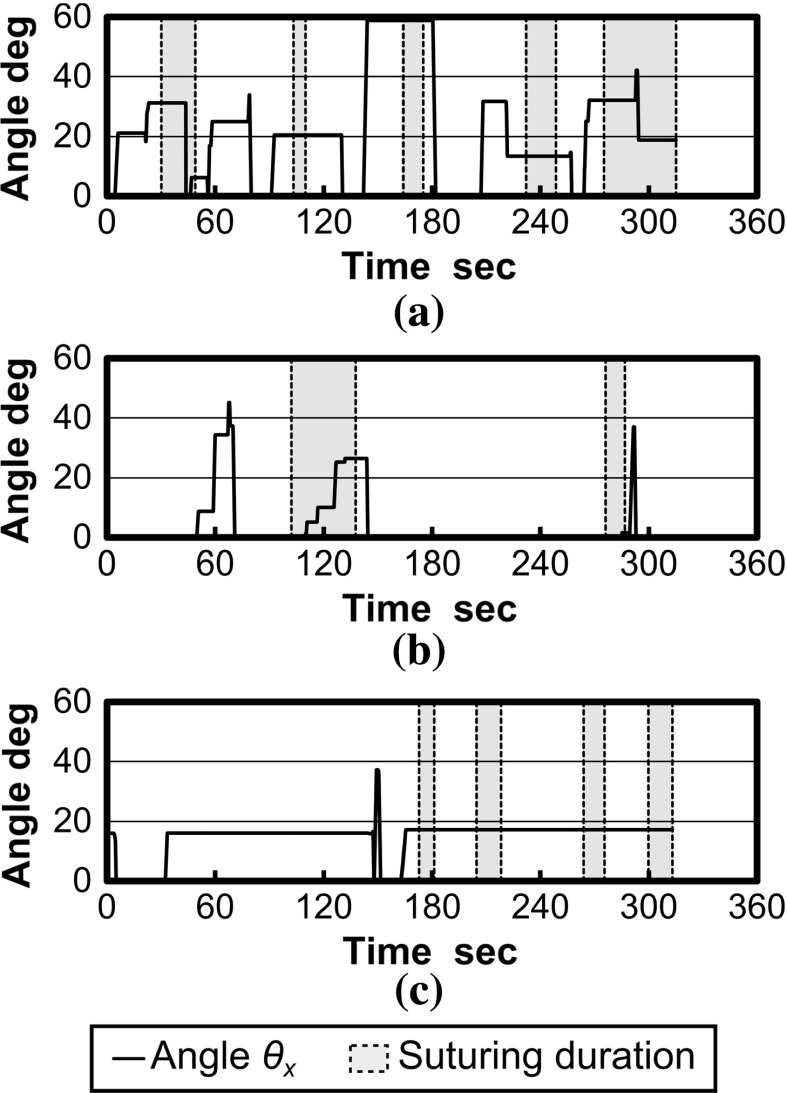
Transition of alternation of angle of viewpoint, **a** Subject 1, **b** Subject 4, **c** Subject 5

Mode and median of pseudo-viewpoint alternation are shown in Table 2. Subjects 2, 5, 6, 8 and 9 stuck to one pseudo- viewpoint so that the mode and median were the same (Fig. 11c). Subject 3, 4 and 7 only used system when inserting or extracting needle so that the mode of the pseudo- viewpoint alternation was 0$^\circ$ (Fig. 11b). The exception was subject 1 who changed pseudo-viewpoint frequently (Fig. 11a).

The number of completed sutures and time taken by each subject to make the first suture is shown in Table 3. Time taken for first suture was longer in five out of nine subjects. It doubled with use of the system for subjects 4 and 8.

No subjects reported existence of latency. Few subjects reported discomfort in needle insertion caused by rendering error on needle. No subject reported existence of rendering error on marks printed on rubber sheet.

## Discussion

We observed an improvement in suturing error with the system. This is because alternation of the viewpoint resulted in an overlooking view which helped depth perception and allowed subjects to aim the marker better. This suggests that the proposed method offers users a sense of reality to the generated view.

We have expected the improvement be greater on the y-axis, but reduction in error was greater on the x-axis. This is because surgeons do not align the position of needle as, for example, an industrial machinery would do; align x-axis first then y-axis next. Instead, surgeons align the position of needle in both axes spontaneously. This suggests that improving spatial perception in one direction could improve task accuracy in both the improved direction and the orthogonal direction.

**Table 2:** Alternation angle of viewpoint

No.	Angle ($^\circ$)	
	Median	Mode
1	20	0
2	23	23
3	0	0
4	0	0
5	16	16
6	25	25
7	2	0
8	15	15
9	21	21
Mean	14	11

**Table 3:** Task performances of the subjects

No.	Error difference w/system$$^{*}$$ (%)	Completed sutures n/2		Time taken for 1st suture$$^{**}$$ (s)	
		w/	w/o	w/	w/o
1	$-$26	5	4	49	73
2	$-$18	1	1	400	307
3	$-$17	1	1	155	288
4	$-$24	2	5	138	53
5	$-$46	4	5	181	45
6	$-$56	6	6	29	32
7	$-$53	1	1	177	314
8	2.1	4	3	151	65
9	$-$1.6	2	1	131	120

*  Negative values are the reduction of suturing error (or the “improvement”) with the use of system. Positive value is the case the error had increased

**  Time taken for first suture is from when the subject picked up the needle to when the whole needle is extracted from the rubber sheet

Experts benefited more from pseudo-viewpoint alternation than novices did. The task prevented inexperienced surgeons from benefiting from pseudo-viewpoint alternation because it was more difficult for them to insert a needle at the desired point than for them to perceive the position for inserting the needle. Because there were only three novices, difference between experts and novices needs to be re-examined after evaluating the system with bigger number of novice subjects.

Inter-group difference observed in this experiment was derived from difference in average skill level in the two groups, not from learning curve. This is because inter-group differences were significant on suturing error both with and without system. More subjects are needed to compare the effect of learning curve.

Average alternation angle was 14$^\circ$. This suggests the ideal viewpoint for suturing should be around 34$^\circ$, which coincides with the accumulated knowledge in laparoscopy.

Rendering error of the needle was problematic, as surgeons perceive needle direction from needle shape. Rendering error on the needles occurred because the current implementation assumed the needle and the forceps are on the same plane as rubber sheet. 3D points for the needle and the forceps were not provided as 3D shape acquisition was not implemented. Rendering errors on the needle and the forceps became large when the planar assumption did not hold.

To apply the system to the clinical field and to improve rendering error on the needle and the forceps, 3D shape acquisition needs to be implemented. There is accumulated knowledge on 3D shape acquisition that is applicable to laparoscopy [10]. Stereopsis-based 3D shape acquisition methods provide dense 3D points without needing modification to the stereo-endoscope. State-of-art stereopsis-based depth acquisition method is reported to run real-time [15]. Since our proposed method was able to run with sparse 3D points, structured light methods are also applicable. Structured light methods require modifications to the endoscope, but are more robust on homogenous surface [16]. These could be further improved by combining segmentation technique to apply needle- or forceps-specific shape acquisition [17].

In the experiment, we substituted 3D shape acquisition by providing system every processing loop a set of uniformly distributed 3D points. We assumed that 3D points are uniformly distributed, but the assumption may not hold for actual clinical cases. Delaunay triangulation is capable providing well-formed meshing even for irregular distribution of points, but assessment is needed to evaluate how the quality of 3D shape acquisition methods affects the overall image quality.

## Conclusion and future work

To allow non-mechanical vision control, we introduced pseudo-viewpoint alternation using image processing. In this paper, we reported the evaluation of the effectiveness of this method by comparing task performances with and without pseudo-viewpoint alternation. A typical suturing task was used in the evaluation. The distance from the center of a dot that defined the ideal needle entry and extraction points was measured as error. With use of our system, error was reduced by 0.37 mm ($p = 0.02$). The improvement had resulted from the change in viewpoint toward an overlooking view that helped depth perception and allowed subjects to aim the markers better. This suggests that the proposed method grants users a sense of reality to the view derived from image processing.

The current system is limited in that 3D shape acquisition was not implemented. In future work, a 3D shape acquisition algorithm will be implemented.
